# Early Presentation of Internal Hernia After Laparoscopic Roux-en-Y Gastric Bypass

**DOI:** 10.7759/cureus.111665

**Published:** 2026-06-28

**Authors:** Faisal Almudayfir, Abdullah S Alzaharani, Abdulaziz Altamran, Mansour Alkhurmudi, Abdulaziz M Tawffeq

**Affiliations:** 1 General Surgery, Prince Sultan Military Medical City, Riyadh, SAU

**Keywords:** bariatric surgery, bowel obstruction, early postoperative complication, gastric bypass, internal hernia, mesenteric defect, mesenteric hernia, revisional bypass, roux-en-y gastric bypass (rygb)

## Abstract

Internal hernia is a potentially serious complication of laparoscopic Roux-en-Y gastric bypass (LRYGB), usually presenting months to years after surgery. Early postoperative internal hernia that occurs within the first week is exceptionally rare and may be difficult to diagnose due to nonspecific symptoms and misleading initial imaging.

We report a case of a 58-year-old female who developed acute epigastric pain and vomiting on the sixth postoperative day following revisional LRYGB with hiatal hernia repair. The initial upper gastrointestinal contrast study showed normal contrast passage. As the symptoms persisted, a CT was done, which demonstrated a markedly dilated biliopancreatic limb with mesenteric swirling, consistent with an internal hernia causing afferent loop obstruction. Emergency exploration revealed herniation of the biliopancreatic limb through a supracolic defect. Reduction and closure resulted in full recovery.

This case highlights the importance of maintaining a high index of suspicion for internal hernia even in the early postoperative period despite appropriate closure of all mesenteric defects.

## Introduction

Obesity is well recognized as a chronic and progressive disease with major global health implications. Excessively overweight individuals are at a higher risk of developing or worsening metabolic chronic diseases and are more prone to poor psychological status. In addition, obesity status can lead to many other adversities affecting the cardiovascular, endocrine, and immune systems. Bariatric surgery, especially laparoscopic sleeve gastrectomy (LSG), remains the most effective long-term treatment modality for achieving durable weight loss and improvement in comorbidities [1].

Revisional procedures are increasingly performed to address weight regain or persistent gastroesophageal reflux following primary weight reduction procedures, commonly LSG. Laparoscopic Roux-en-Y gastric bypass (LRYGB) serves as the preferred revisional procedure of choice [2], consisting of creating a small gastric pouch connected to a Roux jejunal limb with distal jejunojejunostomy forming a Y-shaped intestinal reconstruction.

Internal hernia (IH) is a well-established complication of bariatric surgery, particularly following LRYGB, with a reported incidence of up to 16% [3]. This condition results from iatrogenic mesenteric defects created during surgery, through which a hollow viscus, most commonly the small bowel, may herniate [3]. The most common surgically created defects include Petersen’s space, the jejunojejunostomy mesenteric defect, and the transverse mesocolic defect. Internal hernias typically present one to two years following LRYGB and have been associated with postoperative weight loss and reduction of mesenteric fat, which may contribute to widening of mesenteric spaces [4,5]. In extreme scenarios, IH leads to small bowel obstruction requiring urgent surgical intervention, including reoperation for reduction of the herniated bowel and closure of the mesenteric defect [5]. Although closure of mesenteric defects may reduce the risk of small bowel obstruction or delay the presentation, it does not eliminate the possibility of internal herniation [4-7]. To the best of our knowledge, early postoperative small bowel obstruction due to internal hernia occurring within the first week following revisional LRYGB, despite closure of all standard mesenteric defects, is exceedingly rare and not well described in the literature. This case highlights the importance of maintaining a high index of suspicion for internal hernia even in the immediate postoperative period, even when the mesenteric defects were adequately closed.

## Case presentation

A 58-year-old female with a complex surgical history presented to our clinic with progressive weight regain and persistent gastroesophageal reflux symptoms. Her surgical history included a low anterior resection for rectal cancer 16 years earlier, two pulmonary metastasectomies, and a laparoscopic sleeve gastrectomy performed seven years previously. She had no other known medical comorbidities and was not taking any regular medications. Her family history was negative for obesity, metabolic disorders, and gastrointestinal or colorectal malignancies.

The patient reported progressive weight regain and persistent gastroesophageal reflux symptoms, including retrosternal and epigastric heartburn associated with regurgitation, despite treatment with proton pump inhibitors. At presentation, she weighed 80 kg, with a body mass index (BMI) of 33 kg/m². Preoperative upper gastrointestinal endoscopy revealed Los Angeles grade B esophagitis at the gastroesophageal junction, a sliding hiatal hernia, and a non-*Helicobacter pylori* gastric ulcer. Fluoroscopic evaluation confirmed a large sliding hiatal hernia. Consequently, the patient was scheduled to undergo LRYGB with concomitant hiatal hernia repair.

The patient subsequently underwent LRYGB with hiatal hernia repair. Intraoperatively, dense intra-abdominal adhesions related to the previous colorectal surgery and a large hiatal hernia were encountered. The hernia contents were reduced, and the hiatal defect was repaired by approximating the crura with three continuous stitches using nonabsorbable silk sutures. A 120-cm Roux alimentary limb and a matching 120-cm Y biliopancreatic limb were constructed. An antecolic, isoperistaltic gastrojejunostomy and an isoperistaltic jejunojejunostomy were fashioned, and both anastomoses were confirmed to be leak-free using a methylene blue test. No extensive mesenteric dissection was required. The small bowel was examined from the duodenojejunal junction to the ileocecal valve to ensure that both anastomoses were tension-free.

Prior to mesenteric defect closure, the mesentery was carefully inspected and appeared normal, with no evidence of tension or congestion. Petersen’s space and the jejunojejunal mesenteric defect were meticulously closed using a continuous nonabsorbable silk suture. Although the dimensions of the Petersen’s and jejunojejunostomy mesenteric defects were not formally measured, approximately 15 cm of silk suture was used to close each defect. Esophagopexy was then performed by anchoring the esophagus to the crura with a single nonabsorbable silk suture. No supracolic defect was identified intraoperatively.

The initial postoperative recovery was uneventful. The patient tolerated a liquid diet and passed flatus by postoperative day (POD) 2. An upper gastrointestinal contrast study (upper GI series) performed on POD 3 demonstrated smooth passage of contrast without evidence of obstruction or leak (Figure 1). In our center, an upper GI study is routinely performed after revisional bariatric surgery to assess gastric pouch size and confirm unobstructed passage of contrast through the anastomoses.

**Figure 1:**
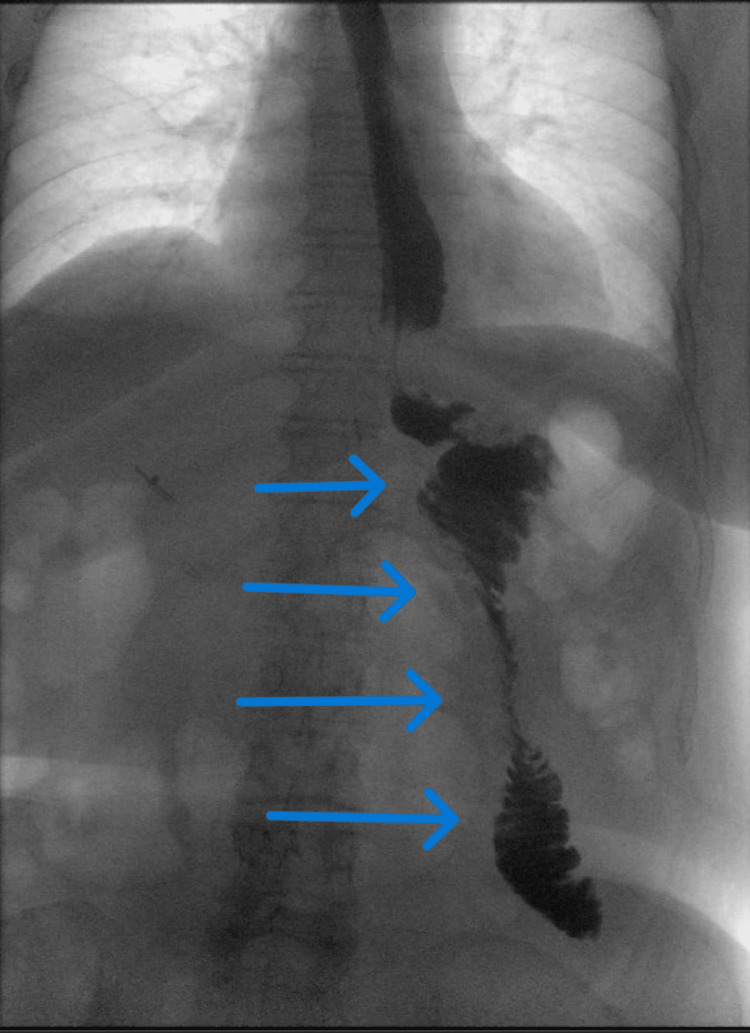
Upper gastrointestinal contrast study demonstrating unobstructed and free passage of contrast through the gastrointestinal tract with no evidence of mechanical obstruction.

The patient passed her first bowel motion on POD 4. However, on POD 6, she suddenly developed severe epigastric pain, nausea, and multiple episodes of bilious vomiting, accompanied by two episodes of brownish loose stool. Despite these symptoms, vital signs and laboratory results remained within normal limits. Abdominal examination revealed only mild epigastric tenderness, with no other significant abnormalities noted on physical examination.

Because vomiting persisted, a contrast-enhanced CT scan was obtained. CT imaging revealed marked dilation of the biliopancreatic limb, a transition point proximal to the jejunojejunal anastomosis, and mesenteric swirling, indicative of internal hernia and evolving afferent loop obstruction (Figures 2-4).

**Figure 2:**
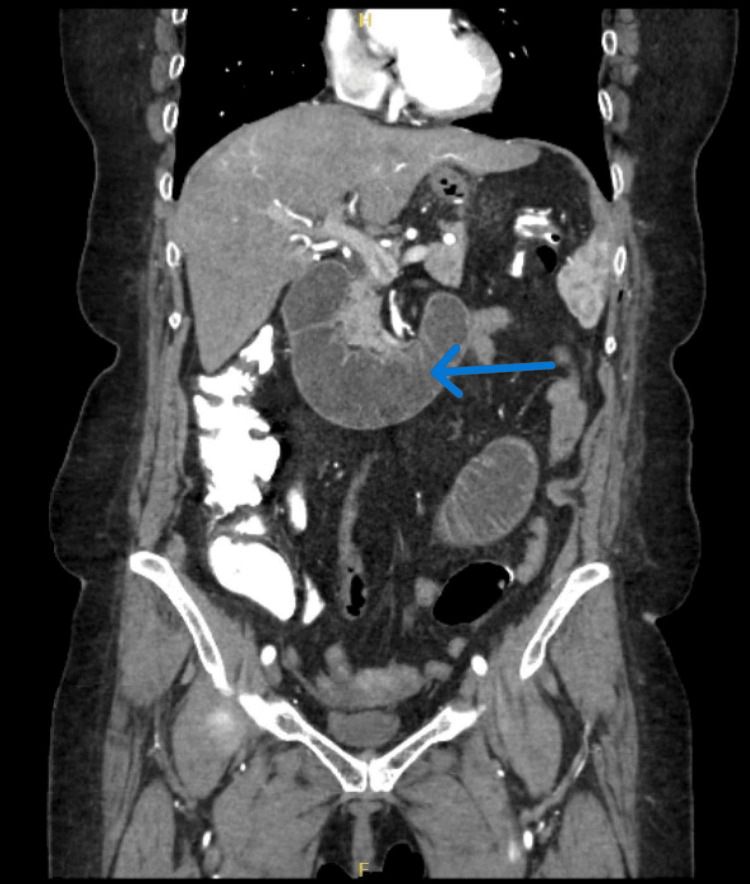
Coronal contrast-enhanced CT scan of the abdomen demonstrating marked dilatation of the biliopancreatic limb (indicated by arrow), suggestive of proximal small bowel obstruction.

**Figure 3:**
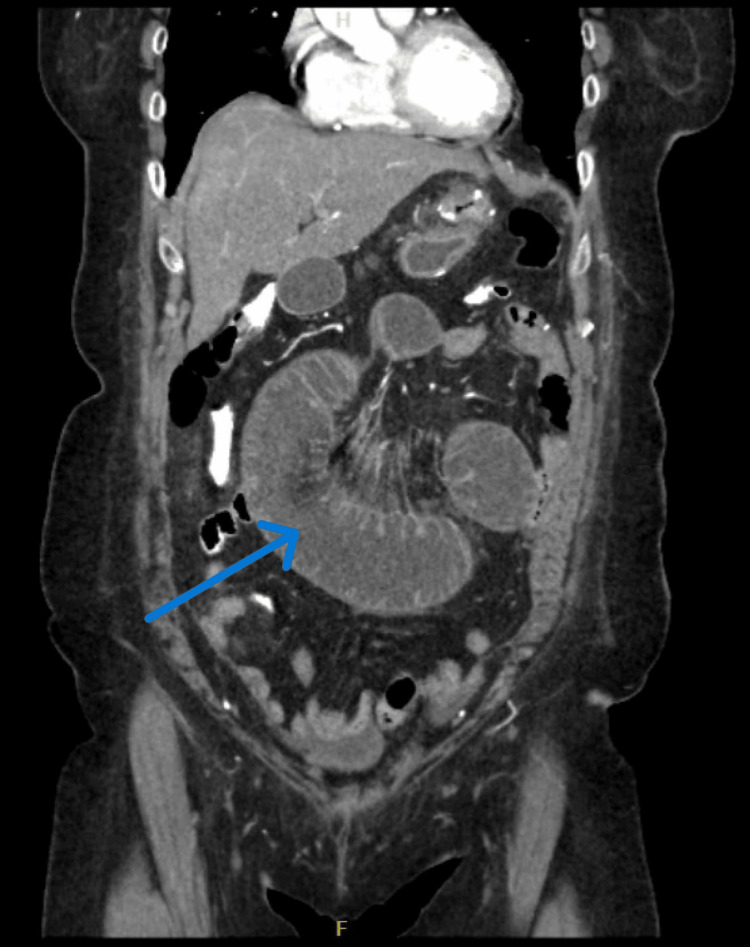
Coronal contrast-enhanced CT scan showing significant dilatation of the biliopancreatic limb (arrow), consistent with an internal hernia and evolving afferent loop obstruction.

**Figure 4:**
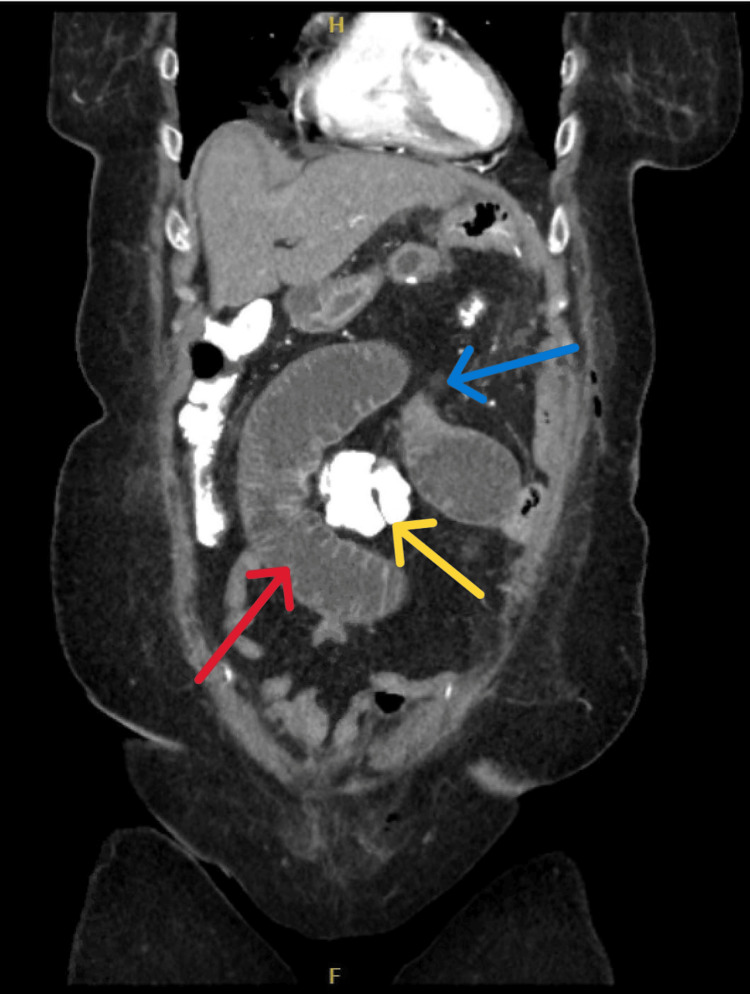
Significant dilation of the biliopancreatic limb with a transition point and mesenteric swirling consistent with internal hernia and afferent loop obstruction. Blue arrow: internal hernia; yellow arrow: transverse colon; red arrow: dilated biliopancreatic limb.

Diagnostic laparoscopy was attempted using a supraumbilical Hasson technique to establish pneumoperitoneum, and a camera port was inserted. Three additional ports were placed under direct vision. However, visualization was limited by markedly dilated bowel loops, and the decision was made to convert to laparotomy.

An upper midline laparotomy, approximately 4-5 cm extending from the xiphoid process, revealed a supracolic internal hernia, with the biliopancreatic limb herniating through a previously unrecognized defect. The herniated biliopancreatic small bowel was pulled from the supracolic defect and returned to the abdominal cavity. The bowel was assessed and found to be viable, with no evidence of ischemia or perforation. The small bowel was run from the duodenojejunal junction to the terminal ileum, confirming appropriate orientation and alignment of the jejunojejunostomy in the left upper quadrant, with unobstructed passage of biliopancreatic content into the common channel. The colon was also inspected and found to be normal, with no additional pathology or free fluid identified. Therefore, the supracolic defect was closed with non-absorbable silk sutures in a 3-5 cm continuous pattern.

The patient recovered uneventfully after the second procedure. Her diet was gradually advanced to clear liquids on POD 2, and she was discharged on POD 5. She was reviewed in the outpatient clinic at two weeks and again at three months postoperatively. At both follow-up visits, she was asymptomatic and tolerating a soft diet and a regular diet, respectively, with no new complaints. She was last seen in clinic one year postoperatively in good general health, with no complaints. At that time, she weighed 68 kg, with a BMI of 28 kg/m². Following Roux-en-Y gastric bypass, she reported complete resolution of gastroesophageal reflux symptoms and was no longer requiring proton pump inhibitor therapy.

## Discussion

Internal hernia is a well-known late complication of LRYGB, resulting from postoperative weight loss and widening of the mesenteric spaces [4,5].

The incidence of internal hernia after LRYGB has been reported with considerable variability in the published literature, ranging from 0.2% to 8.0% within one to three years after surgery [8]. In contrast, Alrubaiaan et al. reported that the incidence of internal hernia after LRYGB could be as high as 16% [3].

Patient-related factors, variations in surgical techniques, and whether the mesenteric defects were closed may have contributed to the wide variation in the reported incidence of internal hernia [4-7].

Three classical defects are typically created due to the natural course of the Roux-en-Y gastric bypass procedure. Petersen’s space is the mesenteric defect located between the mesentery of the Roux limb and the transverse mesocolon or retroperitoneum following gastrojejunostomy. The transverse mesocolic defect is created when the Roux limb is brought through the transverse mesocolon using a retrocolic approach. The jejunojejunostomy (J-J) mesenteric defect is where the mesenteries of the biliopancreatic limb and the Roux limb meet at the level of the jejunojejunostomy.

Closure of Petersen’s and J-J mesenteric defects has been shown to minimize the risk of internal hernia [5]. In fact, a lower reoperation rate for patients with suspected bowel obstruction has been reported among patients who underwent mesenteric defect closure [5]. Our case is a unique presentation, as the prevalence of internal hernia post Roux-en-Y gastric bypass, either as revisional surgery or in patients with significant past surgical abdominal surgeries, is not well established in the literature. These factors may have contributed to the unusually early presentation, occurring within the first postoperative week, despite appropriate closure of the mesenteric defects.

Early postoperative internal hernia causing small bowel obstruction, especially within the first week or month, is rare in occurrence [9]. In a retrospective study evaluating the incidence of early small bowel obstruction (ESBO) within one month following LRYGB, four cases of internal hernia were identified [9]. Patients presented with nonspecific symptoms, including abdominal pain, nausea, vomiting, fever, tachycardia, and shoulder pain [9]. All cases were diagnosed radiologically and required urgent reoperation [9]. In contrast to our case, all four patients had a retrocolic gastrojejunostomy reconstruction [9]. The internal hernias occurred at different anatomical sites: two through the mesocolic window, one through Petersen’s space, and another at the J-J mesenteric defect [9]. Similar to our case, small bowel obstruction requiring reoperation occurred despite closure of all mesenteric defects at the index procedure.

According to a study published by Axer et al., revisional procedures were not only associated with higher rates of intraoperative complications, but have also been noted to be associated with the development of early and late postoperative complications. In addition, patients undergoing revisional bariatric surgery had higher rates of both primary open procedures and conversion from laparoscopic to open surgery [10].

Revisional bariatric surgery presents a unique challenge. Altered anatomy, preexisting adhesions, mesenteric tension, and small or atypical defects created during adhesiolysis may predispose patients to unexpected patterns of herniation. In this case, a supracolic defect (an uncommon location) was responsible, likely related to adhesions and loss of the infracolic compartment following prior colorectal surgery. This highlights that even meticulous closure of standard mesenteric defects does not fully eliminate the risk of early internal hernia.

Diagnostic evaluation of an internal hernia with small bowel obstruction can pose a significant challenge. Symptoms such as nausea, abdominal pain, or vomiting are common in the immediate postoperative period, and early imaging may be falsely reassuring. Upper gastrointestinal contrast studies often fail to demonstrate an internal hernia, particularly in the absence of overt obstruction [11]. CT imaging, however, has characteristic findings, including mesenteric swirl, clustered bowel loops, dilation of the biliopancreatic limb, and transition points, making it the preferred modality for evaluation [11]. Preventive techniques continue to evolve. Running non-absorbable sutures remain standard, but barbed sutures, stapled closures, fibrin glue, and bioabsorbable meshes have been explored as methods to further reduce the risk of internal hernia [12-15]. While these emerging strategies may reduce rates, none have completely prevented the complication.

This case emphasizes the need for a high index of suspicion for internal hernia during the early postoperative period, particularly following revisional bariatric surgery, and reinforces the importance of early CT evaluation when symptoms persist despite initially reassuring findings. Furthermore, it highlights the importance of meticulous closure of all mesenteric defects. However, surgeons should remain aware that even adequate mesenteric defect closure does not completely eliminate the risk of internal herniation.

## Conclusions

Internal hernia can occur even within the first week after LRYGB, despite proper closure of all mesenteric defects. Persistent or unexplained abdominal pain in the early postoperative period warrants careful evaluation, and contrast-enhanced CT should be obtained promptly if symptoms do not resolve. Early recognition and timely operative intervention are crucial to prevent ischemia and ensure optimal patient outcomes.
